# Communication About Microbicide Use Between Couples in KwaZulu-Natal, South Africa

**DOI:** 10.1007/s10461-014-0965-y

**Published:** 2014-12-09

**Authors:** Mitzy Gafos, Robert Pool, Misiwe Adelaide Mzimela, Hlengiwe Beauty Ndlovu, Sheena McCormack, Jonathan Elford, The MDP Team

**Affiliations:** 1 Africa Centre for Health and Population Studies, University of KwaZulu-Natal, Mtubatuba, South Africa; 2 Division of Health Services Research and Management, City University London, London, UK; 3 Medical Research Council Clinical Trials Unit at University College London, Institute of Clinical Trials & Methodology, Aviation House, 125 Kingsway, London, WC2B 6NH UK; 4 Barcelona Centre for International Health Research, University of Barcelona, Barcelona, Spain; 5 Centre for Global Health and Inequality, University of Amsterdam, Amsterdam, Netherlands; 6 Educational Professional Practice Unit, University of Zululand, Kwadlangezwa, South Africa; 7 Department of Nursing Science, University of Zululand, Kwadlangezwa, South Africa

**Keywords:** Microbicides, Adherence, Communication, ‘Disclosure’, South Africa

## Abstract

The ways in which couples communicate about microbicides is likely to influence microbicide uptake and usage. We collected quantitative data about whether women in a microbicide trial discussed microbicides with their partners and explored communication about microbicides during 79 in-depth-interviews with women enrolled in the trial and 17 focus-group discussions with community members. After 4 weeks in the trial, 60 % of 1092 women had discussed microbicides with their partners; in multivariate analysis, this was associated with younger age, clinic of enrolment and not living in households that owned cattle. After 52 weeks, 84 % of women had discussed microbicides; in multivariate analysis, this was associated with not living in households that owned cattle, not living in a household that relied on the cheapest water source, allocation to 0.5 % PRO2000 gel and consistent gel adherence. Qualitative findings highlighted that women in committed relationships were expected to discuss microbicides with their partners and preferred to use microbicides with their partner’s knowledge. Women had different reasons for, and ways of, discussing microbicides and these were influenced by the couple’s decision-making roles. Although there was tolerance for the use of microbicides without a partner’s knowledge, the women who used microbicides secretly appeared to be women who were least able to discuss microbicides. In KwaZulu-Natal, socio-cultural norms informing sexual communication are amenable to microbicide introduction.

## Introduction

The CAPRISA 004 trial in South Africa demonstrated that women assigned to use tenofovir microbicide gel before and after sex had a 39 % lower risk of HIV acquisition when compared to women assigned to use placebo gel [1]. If the on-going FACTS 001 trial confirms the effectiveness of tenofovir gel, vaginal microbicides will be an important additional HIV prevention option for women [2]. The fact that women would be able to use microbicides without their partner’s knowledge was initially perceived as being an important feature of microbicides [3]. However, acceptability research demonstrated that this feature was less important to women in Africa and Asia than it was to women in the United States of America (USA) [4–10].

Research in Sub-Saharan Africa has shown that in many societies women would be expected to discuss microbicides with their partner prior to use. This has been reported in studies evaluating hypothetical willingness to use microbicides [11–15], microbicide acceptability based on the use of surrogate products [16–20], as well as trials evaluating the acceptability of candidate microbicides [5, 21–23]. The exception is women who engage in sex work who would not be expected to discuss microbicides with casual or paying partners [24–27]. Related to this social expectation to discuss microbicides, several of these studies have also shown that women in committed relationships would prefer their partners to be aware of their use of microbicides. This preference appears to be driven by the idea that a couple should not have secrets [28] and a fear of the ramifications if a man discovered his partner using a microbicide without prior discussion [5].

Evidence suggests that the discourse of women’s rights and empowerment that emerged in post-apartheid South Africa distinguishes expectations of sexual communication in South Africa from elsewhere on the continent [29]. In South Africa, there is still an expectation that ideally a couple should discuss microbicides and there is still a preference among women to use microbicides with the knowledge of their partner. However, there appears to be greater tolerance for the use of microbicides without prior discussion in South Africa than in other African countries [30–32]. Indeed, a study in both South Africa and the USA did not find any distinguishable differences between the countries in attitudes towards using a microbicide without prior discussion [8].

A number of early microbicide and diaphragm safety and acceptability studies have reported the number of women who have discussed the use of a gel or diaphragm with their partners in Africa. This has ranged from 75 to 98 % among women and their partners [5, 33–35], 41–50 % among sex workers and their partners and 0–65 % among sex workers and their clients [22, 24, 25]. However, data have not yet been reported on the extent to which women have discussed microbicides with their partners in later stage trials evaluating microbicide effectiveness. As such, there has been no research to date characterising women according to whether they discuss the microbicide with their partner or not. Equally, although it is hypothesised that partner knowledge of microbicides would support adherence, there is limited evidence to support this to date [35]. In the context of a clinical trial, a woman’s decision to discuss the gel with a partner is likely to be influenced by the requirements of participating in a trial, which involves regular study visits often for a year or more. Nonetheless, evidence from clinical trials can provide important information regarding the ways in which women communicate with partners about the use of microbicides [36, 37].

In this paper, we use quantitative and qualitative data collected as part of the Microbicides Development Programme MDP 301 clinical trial at the Africa Centre for Health and Population Studies [38] which is located in the Umkhanyakude district of KwaZulu-Natal, South Africa and was one of six research centres conducting the MDP 301 clinical trial [39–41]. The province of KwaZulu-Natal has the highest HIV prevalence in South Africa at 28 % compared to the national average of 19 % among 15–49 year olds in 2012 [42]. Nationally, HIV incidence among adults was estimated at 1.7 per 100 person years in 2012, but at 2.63 per 100 person years in the Africa Centre for Health and Population Studies population based HIV surveillance programme (2004–2011), peaking at 6.6 per 100 person years in 24 year old women [42, 43]. HIV incidence in the Africa Centre MDP 301 clinical trial cohort was 3.7 per 100 women years (95 % confidence interval 2.5, 5.4) [44]. MDP 301 was a randomised, double-blind, placebo-controlled, phase III clinical trial that evaluated the safety and efficacy of 0.5 % PRO2000 and 2 % PRO2000 microbicide gel in the prevention of vaginally-acquired HIV infection. MDP 301 discontinued the evaluation of the 2 % PRO2000 due to futility in February 2008 on the recommendations of the independent data monitoring committee. In December 2009, MDP 301 announced that 0.5 % PRO2000 had not reduced the risk of HIV acquisition among women. Extensive data were collected on attitudes to microbicides as a HIV prevention option.

In this paper, using quantitative data, we characterize the women who do or do not discuss microbicide use with their partners. Using qualitative data, we examine socio-cultural norms relating to sexual communication and explore the ways in which women discuss microbicide gels with their partners. We explore why and how women use microbicides without their partner’s knowledge, as well as considering the implications of these findings for gel adherence.

## Methods

### Quantitative Methods

The MDP 301 clinical trial has been described in detail elsewhere [39, 40]. In summary, at enrolment, women were randomized to use 2 % PRO2000, 0.5 % PRO2000 or placebo gel until February 2008 when evaluation of 2 % PRO2000 was discontinued due to futility, after which time women were randomised to use 0.5 % PRO2000 or placebo gel [45]. We asked women to insert a pre-filled applicator of gel no more than 1 h before each sex act and to visit the clinics every 4 weeks for 52-weeks.

#### Cohort

In total, 1,177 women enrolled in the Africa Centre MDP 301 clinical trial from March 2006 to August 2008. We included 1,092 women in the analysis, after excluding 85 women who didn’t provide data at the week 4 visit. We enrolled and followed up women at three research clinics: clinic one was located in a township, clinic two was located in a small town, and clinic three was located in a rural area under tribal authority. All three clinics recruited women from rural areas in addition to the immediate locale of the clinic.

#### Dependent Variables

Four weeks after enrolment counsellors administered sexual behaviour questionnaires and collected data about each sex act in the last week, or the last 4 weeks if a woman had not had sex in the last week. For each sex act when gel was used, we asked women the following question: ‘If you used the gel, did you tell your partner about it?’ For the purpose of this analysis, we define women as communicating with their partner about gel use if they informed their partner about using the gel at any single sex act. The outcome variable for this quantitative analysis is talking to the partner about gel use by the week 4 visit. We also assessed which women communicated with their partners about gel use by the end of their trial follow-up (which could be up to 52 weeks).

#### Independent Variables

We considered a range of independent baseline demographic, study specific and socio-economic variables including: age, education, employment, relationship to the head of the household, area of residency, religion, clinic of enrolment, gel randomization group, participation in previous MDP studies, household size (measured using the number of adults who usually sleep in the household divided by the number of rooms usually used for sleeping), water source and fuel source used for cooking, as well as household access to electricity and household ownership of various assets (cattle, a radio, television, telephone, fridge and bicycle). We also assessed associations with behavioural variables collected at the week 4 visit including: sexual activity in the last week, use of reliable contraception (injectable, oral pill, tubal libation or voluntary surgical contraception), condom and gel use in the last week or 4 weeks (measured by dividing the total number of reported sex acts by reported condom or gel use), and impact of gel on sexual pleasure. In the 52-week analysis, we also assessed associations with ‘consistent’ gel use, which was pre-defined in the MDP analysis plan as women reporting gel use during the last sex act for at least 92 % of visits attended; return of at least one used applicator to support their answer when appropriate; and attended at least seven of the expected 13 visits (unless they became pregnant or were infected with HIV during follow-up) [40].

#### Quantitative Analysis

We compared women who reported talking to their partners about gel use by the week 4 visit, to women who did not. We repeated the analysis comparing women who reported talking to their partners about the gel anytime during their participation in the trial, up to a maximum of 52 weeks. We assessed univariate associations using the Pearson χ^2^ test. We tested the contribution to the multivariable model of each variable that was significant in univariate analysis at the 0.10 level using likelihood ratio tests (LRT) [46]. We assessed multivariate associations at the 0.05 level, after controlling for potential confounding factors, through multiple logistic regression analyses. Data were analysed using Stata 10 (StataCorp, College Station, Texas, USA).

### Qualitative Methods

#### Cohort

At enrolment, 101 trial participants were randomly selected to participate in in-depth interviews (IDIs). Of these, 12 women refused to participate mainly due to the time commitment, one woman withdrew from the trial before the first interview, four were never available for interview, and five were not interviewed around the time of their week 4 visit. Consequently, in this analysis we included interviews with 79 trial participants who were interviewed around the time of their week 4 visit. The interview guide has been described elsewhere and included the following topics: partner involvement in decisions relating to sexual matters and partner involvement in gel use [47].

In addition, we advertised focus group discussions (FGDs) at community events and conducted them with women and men who were resident in the trial catchment area but not enrolled in the trial. Community FGDs were stratified by sex, age and area of residence. During the course of the trial 17 standard FGDs were conducted with community members, with an average of nine women or men per group ranging from five to 13. In total six FGDs were conducted with 54 women and 11 FGDs were conducted with 103 men. We also discussed partner involvement in decisions relating to sexual matters in the community FGDs.

Demographics of the IDI and FGD respondents are shown in Table 1.

**Table 1:** Demographics of IDI and FGD participants

	Trial IDIs	Community FGDs (female)	Community FGDs (male)
No of people	79	54	103
No of FGDs	–	6	11
Mean age (range)	34 (19–64)	37 (21–63)	30 (17–67)
Employed (%)	18	13	5
Married (%)^a^	24	41	14

^a^Marital status was ascertained from the IDI & FGD narratives

#### Qualitative Analysis

We conducted IDIs and FGDs in the local language, *isiZulu*. They were audio recorded, transcribed, translated into English, and imported into NVivo 2, later NVivo 8, for coding (NVivo qualitative data analysis software; QSR International Pty Ltd. Version 2, 2002; Version 8, 2008). The majority of transcripts included both the *isiZulu* transcription and English translation, although only the English translation was available for 28 IDIs and four FGDs when direct audio-translation was used with additional quality control of the translated text. Coding was conducted in English. The credibility and trustworthiness of interpretations were considered throughout the trial by presenting results of sub-analyses to local staff and members of the community and participant advisory boards.

We conducted thematic analysis in two stages. Firstly, we analysed the 17 community FGDs coding all text that addressed issues relating to sexual communication. Secondly, we analysed IDIs with 79 women coding all text that addressed issues relating to sexual communication and partner involvement in gel use.

Participants provided written informed consent for trial enrolment. In addition, trial participants and community members provided written informed consent for participation in IDIs and FGDs. The University of KwaZulu-Natal Biomedical Ethics Committee (T111/05) and the South African Medicine Controls Council (N2/19/8/2) reviewed and approved the trial protocol.

## Results

### Quantitative Analysis

Of the 1,092 women included in the analysis, 651 (60 %) women had discussed gel use with their partners by the week 4 visit. Of the 651 who discussed gel with their partners, 578 (89 %) said their partners always knew they were using the gel while 73 (11 %) said their partners sometimes knew they were using it. Women who had discussed gel use with their partners were younger than women who had not. The relationship between discussing gel and age was linear (OR 0.986, *p* value 0.007), although differences between age groups were not statistically significant (*p* value 0.058) (Table 2).

**Table 2:** Individual characteristics of women who discussed gel use with their partner compared to women who did not discuss gel use at week 4

Characteristics	N (%) (col %)	Not discussed N (row %)	Discussed N (row %)	χ^2^ *p* value
	1092 (100 %)	441 (40 %)	651 (60 %)	
Age				
18–24 year olds	309 (28 %)	110 (36 %)	199 (64 %)	0.058
25–34 year olds	224 (21 %)	85 (38 %)	139 (62 %)	
35–44 year olds	265 (24 %)	111 (42 %)	154 (58 %)	
45+ year olds	294 (27 %)	135 (46 %)	159 (54 %)	
Mean age (SD)^a^	35.0 (11.65)	36.2 (11.49)	34.2 (11.70)	0.007
Educational level				
Primary or lower	535 (49 %)	228 (43 %)	307 (57 %)	0.141
Secondary or higher	557 (51 %)	213 (38 %)	344 (62 %)	
Employment status				
Employed	184 (17 %)	81 (44 %)	103 (56 %)	0.270
Unemployed	908 (83 %)	360 (40 %)	548 (60 %)	
Head of household				
Partner	472 (43 %)	208 (44 %)	264 (56 %)	0.090
Parent/in-law	391 (36 %)	142 (36 %)	249 (64 %)	
Self	116 (11 %)	50 (43 %)	66 (57 %)	
Other	113 (10 %)	41 (36 %)	72 (64 %)	
Area of residency				
Rural	857 (78 %)	353 (41 %)	504 (59 %)	0.300
Peri-urban/urban	235 (22 %)	88 (37 %)	147 (63 %)	
Religion				
Zionist	507 (46 %)	202 (40 %)	305 (60 %)	0.882
Shembe	238 (22 %)	104 (39 %)	161 (61 %)	
Christian-mainstream	265 (24 %)	101 (42 %)	137 (58 %)	
Other	82 (8 %)	34 (42 %)	48 (58 %)	
Clinic of enrolment				
Clinic 1—township	419 (39 %)	191 (46 %)	228 (54 %)	0.019
Clinic 2—town	353 (32 %)	128 (36 %)	225 (64 %)	
Clinic 3—tribal authority	320 (29 %)	122 (38 %)	198 (62 %)	
Previous MDP participation^b^				
No	1040 (95.5 %)	425 (41 %)	615 (59 %)	0.086
Yes	49 (4.5 %)	14 (29 %)	35 (71 %)	

^a^ *t* test

^b^Three missing values

As shown in Tables 2, 3 and 4, discussing gel use with a partner was associated at the 10 % level with age (*p* value 0.058), clinic of enrolment (*p* value 0.019), water source (*p* value 0.026), and household ownership of cattle (*p* value 0.021). Although associated at the 10 % level, relationship to the head of household (LRT *p* = 0.694), previous participation in MDP studies (LRT *p* = 0.270), contraceptive use (LRT *p* = 0.251), sexual frequency (LRT *p* = 0.099) and condom use (LRT *p* = 0.177) did not contribute to the model in likelihood ratio tests so were not included in the multivariate model. Table 5 presents the output from the final multivariate model. In the multivariate analysis, older women (AOR 0.98, *p* value 0.006), women enrolled at clinic 1 (AOR 1.00 v clinic 2 at AOR 1.54, *p* value 0.005) and women who lived in households that owned cattle (AOR 0.72, *p* value 0.021) were significantly less likely to have discussed gel use with their partners after 4 weeks in the trial.

**Table 3:** Socio-economic characteristics of women who discussed gel use with their partner compared to women who did not discuss gel use at week 4

Characteristics	N (%) (col %)	Not discussed N (row %)	Discussed N (row %)	χ^2^ *p* value
	1092 (100 %)	441 (40 %)	651 (60 %)	
Water source				
Inside house/yard	333 (30 %)	127 (38 %)	206 (62 %)	0.026
Community source	599 (55 %)	234 (39 %)	365 (61 %)	
Free flowing	160 (15 %)	80 (50 %)	80 (50 %)	
Fuel for cooking				
Electricity	364 (33 %)	143 (39 %)	221 (34 %)	0.856
Gas	88 (8 %)	39 (44 %)	49 (56 %)	
Paraffin	139 (13 %)	57 (41 %)	82 (59 %)	
Wood	501 (46 %)	202 (40 %)	299 (60 %)	
Household ownership (yes)				
Cattle	298 (27 %)	137 (46 %)	161 (54 %)	0.021
Electricity	542 (50 %)	206 (38 %)	336 (62 %)	0.112
Radio	954 (87 %)	385 (40 %)	569 (60 %)	0.960
Television	473 (43 %)	182 (38 %)	291 (62 %)	0.262
Telephone	973 (89 %)	387 (40 %)	586 (60 %)	0.240
Fridge	569 (52 %)	219 (38 %)	350 (62 %)	0.183
Bicycle	181 (17 %)	68 (38 %)	113 (62 %)	0.398

**Table 4:** Sexual behaviour characteristics of women who discussed gel use with their partner compared to women who did not discuss gel use at week 4

Characteristics	N (col %)	Not Discussed N (row %)	Discussed N (row %)	χ^2^ *p* value
	1092 (100 %)	441 (40 %)	651 (60 %)	
Contraceptive use at week 4				
No	403 (37 %)	180 (45 %)	223 (55 %)	0.027
Yes	689 (63 %)	261 (38 %)	428 (62 %)	
Average sex in last week				
3 or less	502 (46 %)	217 (43 %)	285 (57 %)	0.066
4–6	316 (29 %)	129 (41 %)	187 (59 %)	
7 or more	274 (25 %)	95 (35 %)	179 (65 %)	
Condom use in last week/4 weeks				
Always	581 (53 %)	221 (38 %)	360 (62 %)	0.092
Never/sometimes	511 (47 %)	220 (43 %)	291 (57 %)	
Gel use in last week/4 weeks				
Always	1065 (98 %)	428 (40 %)	637 (60 %)	0.405
Never/sometimes	27 (2 %)	13 (48 %)	14 (52 %)	
Gel group				
0.5 %	388 (36 %)	148 (38 %)	240 (62 %)	0.361
2 % PRO 2000	325 (30 %)	141 (43 %)	184 (57 %)	
Placebo	379 (35 %)	152 (40 %)	227 (60 %)	
Impact on sexual pleasure				
Increased	813 (74 %)	336 (41 %)	477 (59 %)	0.278
Same/less	279 (26 %)	105 (38 %)	174 (62 %)

**Table 5:** Multivariate model comparing women who discussed gel use with their partner to women who did not discuss gel use at week 4

	Adjusted odds ratio (95 % CI)	*p* value
Age (mean)	0.98 (0.97, 0.99)	0.006
Clinic of enrolment		
Clinic 1	1.00	
Clinic 2	1.54 (1.14, 2,07)	0.005
Clinic 3	1.32 (0.97, 1.80)	0.076
Water source		
Inside house/yard	1.00	
Community source	1.01 (0.76, 1.36)	0.925
Free flowing	0.70 (0.48, 1.03)	0.074
Household ownership of cattle		
No	1.00	
Yes	0.72 (0.55, 0.95)	0.021

Women who enrolled at clinic 1 in the township were significantly less likely to have discussed gel use with their partner than women who enrolled at clinic 2 in town. To explore possible reasons for differences in clinic of enrolment, we created a variable to identify clinic specific counsellors. In total 13 staff were responsible for gel adherence counselling in the three clinics during this period of observation. We created a binary variable to compare the three main counsellors at clinic 1 to the other 10 counsellors (not presented). When included in the multivariate model, there was no longer a difference between women who discussed gel use with their partners depending on whether they enrolled at clinic 2 (AOR 1.00 95 % CI 0.64, 1.56) or clinic 3 (AOR 0.81 95 % CI 0.50, 1.32) compared to clinic 1. The women counselled by the main three counsellors at clinic 1 were significantly less likely to have discussed the gel with their partners than women counselled by any of the other 10 counsellors at any clinic (AOR 0.56 95 % CI 0.36, 0.88).

We repeated the analysis comparing women who had talked to their partners about the gel to women who had not, any time during their participation in the trial, which was up to a maximum of 52 weeks. By the end of the trial, 84 % (914/1092) of women in this sample had discussed the gel with their partners. Based on univariate associations, the following variables were included in multivariate analysis: water source, cattle ownership, gel randomisation group and consistent gel use. By week 52, 73 % of the women in this sample were defined as consistent gel users. In this model, women who relied on the cheapest water source which was free flowing water such as from a river or stream (AOR 0.53 95 % CI 0.32, 0.89), lived in households that owned cattle (AOR 0.66 95 % CI 0.47, 0.93), and were randomised to 2 % PRO2000 (AOR 0.63 95 % CI 0.42, 0.96) or placebo gel (AOR 0.65 95 % CI 0.44, 0.98, compared to 0.5 % PRO2000) were significantly less likely to have discussed gel with their partners, and women who consistently used gel were significantly more likely than women who had not, to have discussed gel with their partners (AOR 1.51 95 % CI 1.06, 2.14).

Throughout the course of the trial, 31 women reported that it was difficult to insert the gel or inconvenient to use it. In open-ended questions, 12 women reported that the difficulty or inconvenience was due to the fact that they had not told their partners about the gel at the time of use. By week 52, there was a strong association between whether women had discussed the gel with their partners and whether women found it difficult or inconvenient to use gel as only four of these women proceeded to inform their partners (*p* value <0.001). This association remained in the multivariate model at week 52, without substantially changing the other associations (AOR 0.08 95 % CI 0.02, 0.27).

### Qualitative Analysis

Four main themes emerged from the qualitative data: socio-cultural norms of sexual communication, expectations regarding communication about microbicides, women’s experiences of discussing microbicides with their partners, and attitudes and experiences of using microbicides without a partner’s knowledge. We present findings from each of these themes below.

#### Sexual Communication

All respondents in the community FGDs and most of the women in the IDIs had a shared understanding of the traditional norms regarding sexual communication. Within this traditional context, women were not supposed to talk about sex, initiate sex, or even refuse to have sex with their partner. However, there was a palpable schism in opinion about how these traditional norms informed contemporary sexual communication. Approximately half the FGD respondents and about a quarter of IDI respondents, believed that these traditional norms still dominated, while the rest believed that the advent of HIV had altered social expectations regarding sexual communication. The differences were predominately gendered and generational, with most women and younger men believing that communication norms in relationships had changed or were changing.

These shifting expectations were evident in narratives in both the FGDs and IDIs. There was a tension between different expectations regarding the role of women as both submissive and independent. These contradictory expectations appeared to be informed by traditional images of women obeying their husbands, and nationalist images of independent women, which exalt women’s empowerment and promote women’s rights.

Nonetheless, the overwhelming sentiment from the FGDs and IDIs was that in contemporary KwaZulu-Natal, both women and men must break with tradition and talk about sex in response to the HIV epidemic, as this young woman explains:

‘There should be no secrets… It’s not like the olden days. Tell him that there’s something I have found and I will be using it to protect ourselves because no-one wants to die, everybody wants to live, no-one wants to be HIV positive’ (Community FGD, 24 year old woman).

There were risks involved for women who discussed sex. For merely initiating communication about sex, women talked about the risk of abandonment, mistrust, financial marginalisation, verbal conflict or even physical abuse.

#### Expectations Regarding Communication About Microbicides

The vast majority of FGD respondents and more than three quarters of the IDI participants believed that women should discuss gel use with their partners before using it. However, this apparently simplistic statement was not without its complexity, as this quote illustrates:

‘It is important to tell (*uku* *tshela*) your partner about the things that you do ***but only if you know that your partner will agree with you***’ (Trial IDI, 38 year old woman) (*emphasis added*).

Similarly, there were a number of examples in the IDIs where women clearly drew on particular scripts of expected behaviour depending on what they wanted to do. For example, if they wanted to tell their partner about the gel they claimed that culturally they were supposed to talk about sex; alternatively if they did not want to tell their partners about the gel they claimed that culturally they were not supposed to talk about sex. Women at times used these scripts of expected behaviour interchangeably, assigning different priorities to often competing expectations of behaviour depending on the topic.

In the FGDs and IDIs there were different expectations about the form that the discussion about microbicides should take which implied different expectations regarding the decision making process and the role of the male partner. In the FGDs, the main *isiZulu* words used in this context were *imvume, cela, xoxa*, *tshela* and *azisa*. *Imvume* means ‘permission’ and was used in the context of women asking men for permission to use the gel with men being the ultimate decision makers about whether or not women could use gel. *Cela* means to ‘ask’ or ‘negotiate’, *xoxa* means to ‘talk’ or ‘tell someone’ about something, *tshela* means to ‘tell’ or ‘narrate’ or ‘give an account’ of something, and *azisa* means to ‘inform’ but was also used in relation to convincing someone of something. *Cela*, *xoxa*, *tshela* and *azisa* all had an inference of negotiation in the process of seeking agreement.

However, a few mainly younger women used either *tshela* or *azisa* in the sense of literally telling or informing the partner without any expectation of a negotiation or any requirement for permission or consent. In these rarer examples, women were viewed as the ultimate decision makers about whether or not to use gel. The following quote illustrates this perspective but is unusual in coming from a married instead of an unmarried woman:

‘I think I must discuss (*ngi* *phumele* *obala* – speak out or pronounce) so that he will know that I am using this thing (gel). This is my life not his life, I can tell (*ngingam* *tshela*) him that there is something that I am using like this and this, I am protecting myself from the diseases because you are not faithful, I do not know the places you go, you cannot trust a person these days. I can tell (*ngim* *tshele*) him that I am using this thing father (husband) with my life, the life is mine’ (Community FGD, 44 year old woman).

In the IDIs, women most frequently used the word *tshela* to describe their own discussions about the gel with their partners. In addition, woman in the IDIs sometimes used the word *chaza* meaning to explain. The use of this word is understandable in the IDIs, although it never emerged in the FGDs, as women in the trial were describing how they ‘explained’ the gel within the context of the clinical trial.

Throughout the IDIs, there was a sense that the whole discussion hinged on the woman ‘knowing’ her partner and being able to guess his response well enough to find the right words, use the right strategy, at the right time, as this quote demonstrates:

‘A person knows her partner and how he reacts if he is told something’ (Trial IDI, 28 year old woman).

#### Women’s Experiences of Discussing Microbicides

Of the 79 women interviewed at week 4, 56 had talked to their partners about the gel. There were different reasons for talking about the gel, different ways of talking about it, and discussions took place at different times in the process of introducing gel into the relationship.

*Reasons for Talking About Gel* Women offered two main reasons for discussing the gel with their partners. The first reason was that the couple usually discussed sex and the women felt that as the gel would be present during sex, men should be aware of it. The majority of the women interviewed were in long-term stable relationships. Many of the women described these as loving relationships in which they trusted each other and did not have secrets from each other, as stated by this woman:

‘We don’t hide things from each other, he also doesn’t hide anything from me. We usually discuss things before doing them’ (Trial IDI, 22 year old woman).

The second reason, which often overlapped with the first reason, was to avoid conflict if the partner found out about the gel. The concerns they expressed included whether the partner noticed the gel during sex; found the applicators; heard about the gel and suspected his partner was using it; if he had penile problems; or if the gel was found to have safety concerns. Women were also concerned that if their partner felt a difference during sex they may assume the woman was having sex with someone else, which is based on a commonly held myth that men can physically tell during sex if the woman has had sex beforehand.

*Ways of Talking About Gel* Discussion about the use of microbicides took place in different ways. In the majority of cases, women introduced the microbicide study to their partners and then discussed and negotiated the use of the gel. A few women explained that they were with their partners when they first heard about the microbicide study and this triggered the discussion about the potential of the woman joining the study. A few other women described how they simply told their partners about the microbicide study and men accepted their decision to use gel.

The strategies employed by women to discuss the gel with their partners largely depended on the decision-making roles in the relationships. In some examples, it was clear that the ultimate decision of whether or not to use the gel rested with the man. In these cases woman described how they had to convince, cajole and plead with their partners in order to use the gel. In the vast majority of cases, the decision-making was based on a process of negotiation with the aim of reaching joint agreement. In some cases, the decision-making process was on going and involved continuous dialogue, as this quote demonstrates:

‘The first time that I heard from my friends about the study, I sat down with him, talked to him about it, and then he allowed me. Even by the time I came back from the clinic, I as well sat down and informed him about what had been said. I also told him about the gel that there is a preventative thing that they have also given us at the clinic which is in a form of a gel and he asked how it is being used. I then told him. He then said, can I please demonstrate for him how is it being done, I then did as taught, I demonstrated for him’ (Trial IDI, 29 year old woman).

There were other cases where men only provided non-verbal cues. For example, some women talked about the fact that their male partner walked away during the conversation, in which case the woman would assume he was not particularly happy about the gel but was not going to object to her using it. In other cases, women read men’s silence and lack of objection as acceptance of the gel. Throughout most of the narratives, it was evident that women firstly decided whether *they* wanted to use the gel, then opened up discussions with their partners. This appeared to shift the balance of decision-making in the women’s favour.

Less than a quarter of women viewed the decision to use the gel as theirs alone. However, some were willing to state this independence of decision making even when faced with culturally loaded questions, as the exchange below shows:

- **Interviewer**: ‘Does your partner allow (*uyaku* *vumela*) you to insert gel?’
- **Participant**: ‘I insert gel on my own, not because my partner allows (*engi* *vumela*) me to insert’ (Trial IDI, 40 year old woman).

In a few cases, women negotiated their participation in the study with their partner on the basis of only partial information. For example, a few women told their partners they had joined a research study but did not tell them anything about the gel. Other women told their partners that they were using a vaginal gel, although they did not tell them that it was intended as a HIV prevention method. Instead, the gels were described as preventing other sexually transmitted infections, as treatment for vaginal problems, or to prevent cervical cancer. It appeared that by providing partial information, women felt that they could claim they had tried to explain the gel if their partners subsequently challenged them about it. The main reason for only providing partial information appeared to be to avoid having to discuss HIV, which was tied up with issues of trust and fidelity.

*Timing of Talking About Gel* The majority of women who discussed the gel with their partners did so after first learning about the trial at a study screening visit, but before enrolling in the trial. However, about a fifth of women who discussed gel use with partners did so after enrolling in the trial. In some of these cases it appeared that women only wanted to enter into negotiations with their partners after deciding for themselves whether they wanted to join the study and use the gel. While the women who delayed enrolment until after talking to their partner’s may reflect more traditional gendered decision-making roles, women’s decision to discuss gel with their partners only after enrolling in the trial provides another example of the decision-making being shifted to women’s advantage. About a sixth of other women initially used the gel without discussing it with their partners at all and only told them about it when they found study material or noticed a difference during sex.

Although the majority of women told their partners that they were using the gel, few referred to its use every time they had sex. There were three main ways in which gel insertion was managed before sex: (1) the majority of women inserted the gel discretely before sex without telling their partner; (2) a smaller group of women overtly told their partner they were going to insert the gel when passions were roused. Some of these women interpreted their partner’s willingness to wait for them to insert as an act of support. Only a very few women actually inserted the gel in front of their partners and only one woman talked about her partner inserting the gel for her; and (3) a small group of women described how their partners would remind them to insert the gel as a hint for sex, as this quote shows:

‘If he wants sex he just says ‘”gel”, or, “are we ticking” (ticking refers to ticking the coital diary), I know that it is time for sex, so … he has found the easy way to ask for sex’ (Trial IDI, 34 year old woman).

In this way, women described the gel as encouraging communication about sex, which often opened up opportunities for other discussions, for example about condom use. There were numerous examples of women stating that talking about sex and the gel with their husband was more difficult than with an unmarried partner, and that it was especially difficult for women to use the gel without talking to their partner if they were living together.

#### Use of Microbicides Without a Partner’s Knowledge

Although everyone in the FGDs agreed that ideally male partners should know about the gel before it is used, a minority of women and younger male respondents thought that women could be justified in using the gel without their partner’s knowledge in some circumstances. One example was if a woman had experience of her partner refusing specific requests previously. In these circumstances, some women believed that women should use the gel without telling their partner:

‘You do not do something without asking him, you know your partner. You firstly ask him that can I use this or can we use this. If you see that he is not allowing it, you just keep quiet and continue using it secretly’ (Community FGD, 41 year old woman).

Other examples of when use without a partner’s knowledge was justified were if he was HIV positive, had other partners, refused to use condoms, or was frequently drunk, thereby unreliable in terms of condom use. The sense in these examples was that if men failed to be what would traditionally be considered a good and reliable husband, then his partner had the right to breach traditional norms in response to his failings as a husband. In these circumstances, the respondents commented that if the woman was caught using the gel secretly, then she would have to tell her partner the truth. However, they all agreed that this could lead to accusations of infidelity and would cause conflict in the relationship.

After 4 weeks in the trial, 23 of the 79 trial participants interviewed had not discussed their use of the microbicide gel with their partner. All but one of these women had at least one subsequent interview, and by their last interview, 15 had still not discussed gel with their partner (4 at week 24 and 11 at week 52). When talking about using gel without their partner’s knowledge, women mainly referred to using it secretly (*imfihlo*) or hiding it (*fihla*) and described how it was only theirs (not their partners) as in the description of ‘*kuphela ukwazi kwami*’, mine alone.

Four main reasons for women using the gel without telling their partner emerged from the data. Firstly, most of the women still thought it was preferable to discuss gel with their partner and hoped to do so some time in the future but had not yet found the right time. However, of the seven women who subsequently told their partners about the microbicide gel, half only did so after the partner found the applicators or noticed the gel during sex. Secondly, some of the women, mainly young and unmarried, did not think that it was important to discuss the gel with their partners and had no intention of doing so. Thirdly, some women did not want to risk talking to their partners about the gel as they assumed that they would object. This young woman illustrates that by not living in her partner’s house (not being married or cohabitating) she felt she had more ability to decide about the gel:

‘I think he will have a problem, maybe say I should stop the gel, so I thought it is better to continue and hide it from him. He cannot control me because it is my home’ (Trial IDI, 29 year old woman).

Fourthly, about half a dozen women explained that they had not discussed the gel with their partners because they were afraid that they would take their use of gel as a sign of mistrust and respond violently:

‘He doesn’t know about the gel and I don’t want him to know because he is jealous and if he finds out he will beat me. I can’t just talk, I’m afraid of him’ (Trial IDI, 21 year old woman).

Most of the women who were still using gel without their partner’s knowledge after about a month in the study were concerned in case their partners found out about the gel before they had chance to discuss it. However, a few had decided that it was a risk they were willing to take. Using the gel without the knowledge of a stable partner was not viewed as the ideal, but importantly was viewed as possible in some circumstances.

We did not systematically ask women if they would use the gel regardless of their partner’s response. However, of the women who discussed the gel with their partners, approximately a tenth spontaneously reported they would not have used the gel if their partners had objected. An equal number spontaneously reported the opposite, that they would have used the gel even if their partners had objected.

Most women were able to describe strategies that they used to insert the gel before sex, such as going to the toilet to insert the gel if passions were roused. However, it was evident in some women’s accounts that it was more difficult to use the gel when partners were unaware of it, and that inserting additional applicators of gel between sex acts could be particularly difficult.

Only a few women in the IDIs admitted having secondary casual partners and in the main, they had not talked to these partners about the gel. There was no suggestion in the qualitative data that women who did not use condoms were more likely to use the gel without their partner’s knowledge.

## Discussion

In this study, conducted among women enrolled in a microbicide trial in KwaZulu-Natal, more than half the women (60 %) had discussed gel use with their partner by the fourth week of the trial, and the majority (84 %) had discussed gel use by the end of their time in the trial. Our findings, consistent with other evidence, showed that in contemporary KwaZulu-Natal, socio-cultural norms regarding sexual communication are changing in response to the magnitude of the HIV epidemic and the national discourse of women’s rights and gender equality [48, 49]. It is clear from this study, that both traditional gender norms as well as modern ideas of women’s rights inform expectations of communication about microbicides. Women in committed relationships were expected to discuss the use of microbicides with their partners and women clearly preferred to use microbicides with their partner’s knowledge. However, there was tolerance for the use of microbicides without a partner’s knowledge in some circumstances, although the women who used microbicides without their partner’s knowledge appeared to be women least able to discuss microbicides with their partners. Using gel without a partner’s knowledge negatively affected consistent gel adherence over the course of the trial.

From the quantitative data at week 4, older women were less likely to discuss microbicides with their partners than younger women. This may be explained by the idea expressed in the qualitative interviews that traditional views regarding women not being able to talk about sex still prevail. It is possible that older women’s ability to discuss microbicides was limited by the influence of more traditional views among older couples. The qualitative data suggests that the majority of women who had not discussed microbicides with their partners by week 4, still hoped to do so, and many subsequently did. Most women had discussed the use of microbicides by the end of their time in the study and there were no longer any age differences between women who had and had not. As such, the quantitative and qualitative data suggest that older women may be less able to discuss microbicides with their partners initially, but ultimately most find the appropriate time to raise the topic. Although quantitatively younger women were more likely to have discussed the gel with their partners, in the IDIs, the women who expressed no intention or need to discuss gel use with their partners were mainly younger women. These findings highlight the need for microbicide introduction messages to be sensitive to the different needs of younger and older women. Although there is no comparable evidence from microbicide trials, a study in South Africa and Zimbabwe found that older women were less likely to discuss the use of a diaphragm with their partners than younger women [50].

Women who lived in households that relied on free flowing water and owned cattle were also less likely to discuss microbicides with their partners. The significance of water source diminished in the multivariate model at week 4 but remained at week 52, whereas the association with owning cattle was found at both weeks 4 and 52. Water source and household assets were included in this analysis as socio-economic determinants. However, the qualitative data hint at an alternative interpretation of the relevance of these variables suggesting that in addition to indicating household wealth, they may also indicate traditional status. Cattle are the most important symbol of status in Zulu culture and are the basis of marital arrangements [51]. While the use of free flowing water is usually an indicator of lower socio-economic status, in this area it also suggests that the household is particularly remote [52]. On the basis of the discussions about traditional versus modern views about communication, we could speculate that cattle ownership and the use of free flowing water, in this analysis, are signs of more traditional households in which it is more difficult for women to talk about microbicides.

At week 4, we measured gel adherence in the last week based purely on self-reported usage, which in known to be subject to social-desirability bias [41]. After only four weeks of using gel, adherence was not associated with whether a woman had discussed the gel with her partner or not. However, over the course of the trial we used a longer-term measure of adherence, which was based on self-reported use at multiple time points, clinic attendance, and the return of used applicators. By the end of the trial, women who had discussed gel with their partners were 50 % more likely to have consistently used gel than women who had not. This is a striking and important difference, which confirms the assumption that using the gel without a partner’s knowledge may hinder adherence for some women [20, 53]. The qualitative results also support this finding by highlighting that it was more difficult for some women to use gel without their partner’s knowledge, especially between sex acts. However, it is important to note that although the longitudinal measure of adherence provided a more complete picture of women’s non-use of gel than cross sectional measures, it was still constrained by relying on self-reported or proxy measures and may well over estimate true adherence. It will be important to explore the association between women’s communication with their partner’s and usage using objective measures of adherence in future trials.

This study reflects microbicide communication between stable couples, many of which in in-depth interviews described their relationships as loving and trusting, and as such is likely to be substantially different from women in less stable unions. However, among women in stable relationships, the qualitative findings suggest that it would be more difficult for women to use microbicides without their partner’s knowledge if they were married or living together. There were no differences in the proportion of women who discussed microbicides with their partners based on their relationship to the head of the household, although we were not able to control for marital status or cohabitation with a partner in the quantitative analysis. Not cohabitating has been shown to be associated with using a diaphragm without a partner’s knowledge [54].

The quantitative data showed that the proportion of women who discussed microbicides with their partners differed between the clinics at week 4, but not by the end of the trial. Although the counselling scripts were supposed to be standardised, this difference appears to be related to specific counsellors. This may reflect the individual opinions of counsellors or differing levels of competency in counselling. The impact of counselling on women’s decision to discuss microbicides with their partners has not been measured previously in microbicide or diaphragm trials. This finding suggests that the counselling process is likely to influence women’s decision or ability to discuss microbicides with their partners. The qualitative data showed that women were creative in their approaches to discussing microbicides, providing as much or as little information as they felt was warranted depending on their knowledge of their partners character. The use of partial information was also reported by women negotiating the use of diaphragms [54]. The impact on adherence of a partner knowing about the microbicide, the importance of counselling on women’s ability to discuss microbicides, and the diversity of strategies that women employ to discuss microbicides, highlight the need for more focus on the development and evaluation of counselling messages that are tailored to women’s individual circumstances. In acknowledging women’s preference for discussing microbicides with their partners there is increasing focus on the need to include men in microbicide introduction programmes [28]. However, as this analysis suggestions, it is important to ensure that by including men, we don’t undermine women’s ability to make autonomous decisions about whether to discuss microbicides with their partners and to employ creative strategies regarding how to discuss them.

By the end of the trial, women differed in terms of whether they had discussed microbicides with their partners depending on the type of gel they had been allocated to use. There is no indication in this study or any other MDP analysis that women preferred any one of the three gels used in the trial [45, 55]. The majority of women assigned to 2 % PRO2000 gel stopped using the gel before the end of their year follow up period due to the closure of this gel arm, and therefore would have had less time to discuss the gel with their partners. However, this is not a sufficient explanation, as it does not explain the difference for the placebo group. As such, it is not clear why women assigned to the 2 % PRO2000 or placebo gel groups would be less inclined to talk to their partners about the gel than women given 0.5 % PRO2000 in this blinded trial. One of the few studies to have reported on women’s communication by gel type, compared three different formulations of N-9 and found no correlation between women’s preferences for formulations and discussing the product with their partners [5].

To date, the later stage microbicide effectiveness trials have not reported on the extent to which women discuss microbicide gels with their partners. A diaphragm trial found that 15 % and 19 % of women in Durban and Johannesburg respectively used the diaphragm without their partner’s knowledge for up to 2 years, compared to only 1 % of women in Zimbabwe. The authors concluded that ‘women in South Africa seemed to emphasize individual rights and personal agency to justify covert use as compared to women from Zimbabwe, who made a stronger case about negative consequences if caught’ (54;1552). This conclusion infers a link between women’s agency and diaphragm use without a partner’s knowledge. Our findings add additional nuance to this debate of whether it is women’s personal agency or lack thereof, which would encourage women to use a microbicide or diaphragm secretly. On the one hand, the qualitative data, especially from the IDIs with trial participants, suggest that the growing national discourse that women have a right to protect themselves from HIV is viewed as justification to use microbicides without a partner’s knowledge. On the other hand, the quantitative and qualitative findings suggest that women who used the gel without their partner’s knowledge were the ones who felt least able to discuss microbicides with their partners. As such, in this predominantly rural area of KwaZulu-Natal it is not clear the extent to which women’s agency increases women’s ability to discuss microbicides with their partner or use microbicides without their knowledge. The qualitative data suggests that women’s decision about if, when and how to discuss microbicides with their partners is influenced by the gendered decision-making roles in the relationship. The importance of gender-norms and decision-making roles for HIV prevention [56–59] and microbicide acceptability is well recognised [28, 31, 60], although rarely measured in microbicide trials. In terms of supporting women to use microbicides in the future, we need to further explore women’s decisions of whether or not to discuss microbicides with their partners, and the role that gender-norms and decision-making roles play in this decision. It is however also important to note, that the ways in which women reported if, when and how they communicated with their partners about microbicides, could also be influenced by social-desirability and the dichotomous discourse of the role of traditional verses modern women in South Africa.

In addition to exploring how decision-making roles influence women’s discussion about microbicides with their partners, it will be equally important to assess how the availability of microbicides influences decision-making roles within couples. The qualitative data provided a number of examples whereby decision-making power was shifted in the women’s favour by the fact that these were female-used products. The most important factor, as seen with diaphragms previously [54], is that unlike with condoms, once microbicides have been discussed by a couple, most women then proceed to use them without needing to negotiate their use every time they have sex. However, despite the overarching sentiment that women had the right to use a microbicide to protect themselves from HIV with or without a partner’s knowledge, the qualitative data also demonstrated that the implications of male partner’s finding out about women’s use of microbicides could be considerable. It is evident from these findings that male partner’s opposition to microbicides and the risks involved with using microbicides without a partner’s knowledge will remain a barrier to microbicide access for some women. Given the high levels of intimate partner violence in South Africa, messaging around microbicides needs to take account of the risks inherent in the secret use of microbicides for some women [61].

In this study, women’s discussions with their partners about enrolling in the clinical trial and using a microbicide gel were merged into one conversation. It was impossible to separate these conversations as for women they were one and the same. Similarly, in this blinded trial it was impossible to discern what impact women’s chance of randomisation to a placebo gel had on the decision to discuss microbicides. The reasons for discussing microbicides in the future when they are known to prevent HIV acquisition and are widely available, are likely to be different to the reasons for discussing microbicides in the context of a placebo-controlled clinical trial. Nonetheless, these findings provide an important insight into some of the challenges that women face when discussing microbicides with their partner and the creativity that women are likely to employ in their use of microbicides [36, 37].

## Conclusion

The women enrolled in this trial, conducted in KwaZulu-Natal, clearly preferred to use microbicides with their partner’s knowledge. Changing attitudes to gender relations that have been influenced by the national discourse on women’s rights and the HIV epidemic, appear to facilitate female initiated conversations about sex, and enable women to talk about microbicides. Traditional ideas of women’s roles are still prevalent, and inhibit some women from discussing microbicides with their partner, although this analysis demonstrates that the majority of women are successful at negotiating their use or feel justified in using microbicides without a partner’s knowledge. While there is tolerance for the use of microbicides without a partner’s knowledge, and a minority of women succeed in using the gel secretly, this appears to have a negative impact on long-term gel adherence. Introductory microbicide programmes will rely on clear and consistent counselling messages, that should be tailored to women’s specific circumstances. Our findings demonstrate that in KwaZulu-Natal the socio-cultural norms relating to sexual communication are amenable to the introduction of microbicides. The findings also present yet another example of the important contribution that microbicides can make in terms of offering women HIV prevention options that they can both use and control.

## References

[1] Abdool-Karim Q, Abdool-Karim SS, Frohlich JA (2010). Effectiveness and safety of tenofovir gel, an antiretroviral microbicide, for the prevention of HIV infection in women. Science.

[2] FACTS. Facts Consortium Johannesburg: Follow-on African Cosortium for Tenofovir Studies (FACTS); Available at: http://www.facts-consortium.co.za/. [Accessed 2nd February 2012].

[3] Koo HA, Woodsong C, Dalberth B, Viswanathan M, Simons-Rudolph A (2005). Context of acceptability of topical microbicides: sexual relationships. J Soc Issues.

[4] Carballo-Diéguez A, Balan IC, Morrow K (2007). Acceptability of tenofovir gel as a vaginal microbicide by US male participants in a Phase I clinical trial (HPTN 050). AIDS Care.

[5] Coggins C, Elias CJ, Atisook R (1998). Women’s preferences regarding the formulation of over-the-counter vaginal spermicides. AIDS..

[6] El-Sadr WM, Mayer KH, Maslankowski L (2006). Safety and acceptability of cellulose sulfate as a vaginal microbicide in HIV-infected women. AIDS..

[7] Hoffman S, Morrow KM, Mantell JE, Rosen RK, Carballo-Diéguez A, Gai F (2010). Covert use, vaginal lubrication, and sexual pleasure: a qualitative study of urban U.S. Women in a vaginal microbicide clinical trial. Arch Sex Behav.

[8] Morrow K, Rosen R, Richter L (2003). The acceptability of an investigational vaginal microbicide, PRO 2000 gel, among women in a phase I clinical trial. J Womens Health (Larchmt).

[9] Rosen RK, Morrow KM, Carballo-Diéguez A (2008). Acceptability of tenofovir gel as a vaginal microbicide among women in a phase I trial: a mixed-methods study. J Womens Health (Larchmt)..

[10] Carballo-Diéguez A, Giguere R, Dolezal C (2011). “Tell Juliana”: acceptability of the candidate microbicide VivaGel(R) and two placebo gels among ethnically diverse, sexually active young women participating in a phase 1 microbicide study. AIDS Behav.

[11] Veldhuijzen N, Nyinawabega J, Umulisa M (2006). Preparing for microbicide trials in Rwanda: focus group discussions with Rwandan women and men. Cult Health Sex.

[12] Ramjee G, Gouws E, Andrews A, Myer L, Weber A (2001). The acceptability of a vaginal microbicide among South African men. Int Fam Plan Perspect.

[13] van de Wijgert JH, Khumalo-Sakutukwa GN, Coggins C (1999). Men’s attitudes toward vaginal microbicides and microbicide trials in Zimbabwe. Int Fam Plan Perspect.

[14] Coggins C, Blanchard K, Friedland B (2000). Men’s attitudes towards a potential vaginal microbicide in Zimbabwe, Mexico and the USA. Reprod Health Matters..

[15] Bisika T (2009). Potential acceptability of microbicides in HIV prevention in stable marital relationships in Malawi. J Fam Plann Reprod Health Care.

[16] Salter ML, Go VF, Celentano DD (2008). The role of men in women’s acceptance of an intravaginal gel in a randomized clinical trial in Blantyre, Malawi: a qualitative and quantitative analysis. AIDS Care.

[17] Jones DL, Weiss SM, Chitalu N, Bwalya V, Villar O (2008). Acceptability of microbicidal surrogates among Zambian women. Sex Transm Dis..

[18] Green G, Pool R, Harrison S (2001). Female control of sexuality: illusion or reality? Use of vaginal products in south west Uganda. Soc Sci Med.

[19] Pool R, Whitworth JA, Green G, Mbonye AK, Harrison S, Wilkinson J, Hart GJ (2000). An acceptability study of female-controlled methods of protection against HIV and STDs in south-western Uganda. Int J STD AIDS..

[20] Montgomery CM, Lees S, Stadler J (2008). The role of partnership dynamics in determining the acceptability of condoms and microbicides. AIDS Care.

[21] Bentley ME, Fullem AM, Tolley EE (2004). Acceptability of a microbicide among women and their partners in a 4-country phase I trial. Am J Public Health.

[22] Turner AN, Van Damme K, Jamieson DJ (2009). Predictors of adherent use of diaphragms and microbicide gel in a four-arm, randomized pilot study among female sex workers in Madagascar. Sex Transm Dis.

[23] Montgomery CM, Gafos M, Lees S (2010). Re-framing microbicide acceptability: findings from the MDP301 trial. Cult Health Sex.

[24] Rustomjee R, Abdool Karim Q, Abdool Karim SS, Laga M, Stein Z (1999). Phase 1 trial of nonoxynol-9 film among sex workers in South Africa. AIDS..

[25] Vandebosch A, Goetghebeur E, Ramjee G (2004). Acceptability of COL-1492, a vaginal gel, among sex workers in one Asian and three African cities. Sex Transm Infect.

[26] Greene E, Batona G, Hallad J (2010). Acceptability and adherence of a candidate microbicide gel among high-risk women in Africa and India. Cult Health Sex.

[27] Visness CM, Ulin P, Pfannenschmidt S, Zekeng L (1998). Views of Cameroonian sex workers on a woman-controlled method of contraception and disease protection. Int J STD AIDS.

[28] Woodsong C, Alleman P (2008). Sexual pleasure, gender power and microbicide acceptability in Zimbabwe and Malawi. AIDS Educ Prev.

[29] Mantell JE, Needham SL, Smit JA (2009). Gender norms in South Africa: implications for HIV and pregnancy prevention among African and Indian women students at a South African tertiary institution. Cult Health Sex..

[30] Orner P, Harries J, Cooper D (2006). Challenges to microbicide introduction in South Africa. Soc Sci Med.

[31] MacPhail C, Terris-Prestholt F, Kumaranayake L (2009). Managing men: women’s dilemmas about overt and covert use of barrier methods for HIV prevention. Cult Health Sex.

[32] Ramjee G, Morar NS, Braunstein S (2007). Acceptability of Carraguard, a candidate microbicide and methyl cellulose placebo vaginal gels among HIV-positive women and men in Durban, South Africa. AIDS Res Ther.

[33] Carraguard (2010). Expanded safety and acceptability of the candidate vaginal microbicide Carraguard(R) in South Africa. Contraception.

[34] Hira SK, Spruyt AB, Feldblum PJ (1995). Spermicide acceptability among patients at a sexually transmitted disease clinic in Zambia. Am J Public Health.

[35] van der Straten A, Moore J, Napierala S (2008). Consistent use of a combination product versus a single product in a safety trial of the diaphragm and microbicide in Harare, Zimbabwe. Contraception.

[36] Woodsong C, MacQueen K, Amico KR (2013). Microbicide clinical trial adherence: insights for introduction. J Int AIDS Soc.

[37] Tolley E, Friedland B, Gafos M. Socioeconomic and behavioural factors influencing choice, adherence and success of microbicide formulations. In: José das Neves BS, editor. Drug delivery and development of anti-HIV microbicides 2014.

[38] Tanser F, Hosegood V, Bärnighausen T (2008). Cohort profile: Africa Centre Demographic Information System (ACDIS) and population-based HIV survey. Int J Epidemiol.

[39] Nunn A, McCormack S, Crook AM (2009). Microbicides development programme: design of a phase III trial to measure the efficacy of the vaginal microbicide PRO 2000/5 for HIV prevention. Trials.

[40] McCormack S, Ramjee G, Kamali A (2010). PRO2000 vaginal gel for prevention of HIV-1 infection (Microbicides Development Programme 301): a phase 3, randomised, double-blind, parallel-group trial. Lancet.

[41] Pool R, Montgomery CM, Morar NS (2010). A mixed methods and triangulation model for increasing the accuracy of adherence and sexual behaviour data: the Microbicides Development Programme. PLoS One.

[42] Shisana O, Rehle T, Simbayi LC, Zuma K, Jooste S, Zungu N, Labadarios D, Onoya D (2014). South African National HIV prevalence, incidence and behaviour survey.

[43] Tanser F, Bärnighausen T, Grapsa E, Zaidi J, Newell M-L (2013). High coverage of ART associated with decline in risk of HIV acquisition in rural KwaZulu-Natal, South Africa. Science.

[44] Crook A, Gilbert, C., Birger, R. Final report for MDP 301 Centre Report. 2009.

[45] Gafos M, Mzimela M, Ndlovu H (2011). “One teabag is better than four”: Participants response to the discontinuation of 2% PRO2000/5 microbicide gel in KwaZulu-Natal, South Africa. PLoS One.

[46] Kirkwood B, Sterne J (2003). Essential medical statistics.

[47] Gafos M, Pool R, Mzimela MA (2014). The implications of post-coital intravaginal cleansing for the introduction of vaginal microbicides in South Africa. AIDS Behav.

[48] Susser I (2009). AIDS, sex, and culture: global politics and survival in Southern Africa.

[49] Ndinda C, Uzodike UO, Chimbwete C, Pool R (2007). Gender relations in the context of HIV/AIDS in rural South Africa. AIDS Care.

[50] Sahin-Hodoglugil NN, Montgomery E, Kacanek D (2011). User experiences and acceptability attributes of the diaphragm and lubricant gel in an HIV prevention trial in southern Africa. AIDS Care.

[51] Berglund A-I (1976). Zulu thought-patterns and symbolism.

[52] Muhwava W (2007). Trends of economic status of households in the ACDIS.

[53] Montgomery ET, van der Straten A, Chidanyika A (2011). The importance of male partner involvement for women’s acceptability and adherence to female-initiated HIV prevention methods in Zimbabwe. AIDS Behav.

[54] Sahin-Hodoglugil NN, van der Straten A, Cheng H (2009). Degrees of disclosure: a study of women’s covert use of the diaphragm in an HIV prevention trial in sub-Saharan Africa. Soc Sci Med.

[55] Gafos M, Mzimela M, Sukazi S (2010). Intravaginal insertion in KwaZulu-Natal: sexual practices and preferences in the context of microbicide gel use. Cult Health Sex.

[56] Jewkes R, Nduna M, Levin J (2008). Impact of stepping stones on incidence of HIV and HSV-2 and sexual behaviour in rural South Africa: cluster randomised controlled trial. BMJ.

[57] Jewkes R, Morrell R (2010). Gender and sexuality: emerging perspectives from the heterosexual epidemic in South Africa and implications for HIV risk and prevention. J Int AIDS Soc.

[58] Pettifor AE, Measham DM, Rees HV, Padian NS (2004). Sexual power and HIV risk, South Africa. Emerg Infect Dis.

[59] Fladseth K, Gafos M, Newell M, McGrath N. More equitable gender norms are associated with higher levels of condom use at last sex act among HIV-positive men and women: rural KwaZulu-Natal, South Africa. South African AIDS Conference; 18–21 June 2013; Durban, South Africa 2013.

[60] Mantell JE, Dworkin SL, Exner TM (2006). The promises and limitations of female-initiated methods of HIV/STI protection. Soc Sci Med.

[61] Jewkes RK, Dunkle K, Nduna M, Shai N (2010). Intimate partner violence, relationship power inequity, and incidence of HIV infection in young women in South Africa: a cohort study. Lancet.

